# Identification of *HLA-A*, *HLA-B*, and *HLA-C* triple homozygous and double homozygous donors: a path toward synthetic superdonor advanced therapeutic medicinal products

**DOI:** 10.3389/fimmu.2025.1626787

**Published:** 2025-09-16

**Authors:** Daniel Naumovas, Barbara Rojas-Araya, Catalina M. Polanco, Victor Andrade, Rita Čekauskienė, Beatričė Valatkaitė-Rakštienė, Inga Laurinaitytė, Artūras Jakubauskas, Mindaugas Stoškus, Laimonas Griškevičius, Ivan Nalvarte, Jose Inzunza, Daiva Baltriukienė, Jonathan Arias

**Affiliations:** ^1^ Laboratory of Nuclease Enabled Cell Therapies, Vilnius University Life Science Center EMBL Partnership Institute for Gene Editing Technologies, Vilnius, Lithuania; ^2^ Vilnius Santaros Klinikos Biobank, Vilnius University Hospital Santaros Klinikos, Vilnius, Lithuania; ^3^ Department of Molecular Medicine; Hematology, Oncology and Transfusion Medicine Center, Vilnius University Hospital Santaros Klinikos, Vilnius, Lithuania; ^4^ Division of Neurogenetics and Molecular Psychiatry, Department of Psychiatry and Psychotherapy, Medical Faculty, University of Cologne, Cologne, Germany; ^5^ Department of Cognitive Disorders and Old Age Psychiatry, University Hospital Bonn, Bonn, Germany; ^6^ Karolinska Institutet, Department of Neurobiology, Care Sciences and Society, BioClinicum, Solna, Sweden; ^7^ Karolinska Institutet Stem Cell Organoid (KISCO) facility, Department of Laboratory Medicine, Huddinge, Sweden; ^8^ Karolinska Institutet, Department of Laboratory Medicine, Huddinge, Sweden; ^9^ Department of Biological Models, Institute of Biochemistry, Life Sciences Center, Vilnius University, Vilnius, Lithuania

**Keywords:** superdonor, HLA class I, immune compatibility, hypoimmunogenic, population genetics

## Abstract

Human-induced pluripotent stem cells with broad immune compatibility are highly desirable for regenerative medicine applications. Human leukocyte antigen (HLA) class I homozygous cell sources are ideal for immune compatibility modeling. Here, we profile *HLA-A*, *HLA-B*, and *HLA-C* alleles in 3,496 Lithuanian donors genotyped at three-field resolution. The five most frequent alleles constitute 74.6% of *HLA-A*, 43.2% of *HLA-B*, and 59.2% of *HLA-C*, with HLA-A*02:01:01, HLA-B*07:02:01, and HLA-C*07:02:01 being the most common. Lithuanian allele frequencies closely resemble those of European-American and British populations. We identified 153 double homozygotes and 51 triple homozygotes for *HLA-A*, *HLA-B*, and *HLA-C*. Compatibility modeling showed that triple homozygous profiles match 60.5% of Lithuanians, 13.4% of the British population, and 7.4% of European-Americans. CRISPR-Cas9 guide RNA design yielded 54 candidates predicted to disrupt *HLA-A* or *HLA-B* while preserving *HLA-C*, producing edited profiles matching over 97.9% of Lithuanians, 95.7% of European-Americans, and 95.5% of the British population. Finally, we established 15 fibroblast lines from triple homozygotes as a bioresource for the derivation of human-induced pluripotent stem cells and immune compatibility studies.

## Introduction

Transplantation of allogeneic organs, tissues, and cells is constrained by immune matching between the graft and the host. Immune matching is mediated by the human leukocyte antigen (HLA) genes. These genes are clustered in a 3.7-Mbp locus on chromosome 6, are highly polymorphic, and their inheritance is reported as having intermediate linkage disequilibrium (1, 2). The recent adoption of high-resolution haplotyping in clinics has improved the accuracy of immune matching for the more than 42,000 HLA alleles cataloged in the IPD-IMGT/HLA Database (3). Pursuing a high level of matching is intended to minimize adverse events, such as graft-versus-host disease (GVHD) or immune rejection, which are frequently managed with immunosuppressive drugs. A broad assortment of immunosuppressive treatments is available for the management of transplantation, encompassing small molecule inhibitors, antimetabolites, corticosteroids, and antibodies (4–6). However, immunosuppressive therapies are associated with an increased risk of infection (7, 8). Therefore, pursuing a high level of matching is intended to minimize adverse events caused by immune rejection and immune suppression. The importance of a high degree of HLA immune matching for improving survival rates is well documented in the literature (9, 10) for exemplary primary cell types, and it is highly desirable for induced pluripotent stem (iPS) cell-based applications.

HLA class I homozygous individuals offer increased immune compatibility with a relatively larger portion of the population. They are very scarcely represented, as expected from Mendelian ratios. Cells from naturally occurring triple and double homozygous individuals are very valuable for the study of immune compatibility and applications of regenerative medicine.

Genome editing tools are currently used to engineer synthetic immune compatibility, also called hypoimmunogenicity. This aids in overcoming the challenges of identifying rare haplotypes in donor pools. Several approaches have been developed to bypass immune recognition by cytotoxic T cells while retaining self-recognition mediated by NK cells. The most frequent loss-of-function strategies include the knockout of specific HLA class I (11) and class II genes, beta-2-microglobulin (*B2M*) (12, 13), *CIITA* (14), *TAP1* or *TAP2*, and *CD74* (15). Conversely, the most frequent gain-of-function strategies involve the knock-in of *CD47* and *HLA-E* (16). Pioneering studies have demonstrated that gene-editing depletion of *HLA-A* and *HLA-B* genes preserves host NK cell recognition while preventing CD8 T-cell mediated host-versus-graft rejection (17). This approach yields cells currently known as *HLA-C* retained. Triple and double homozygous samples are an ideal cell source for modulating immunogenicity, as they start from a relatively higher level of immune compatibility. Furthermore, they can be engineered in their *HLA* genes using programmable nucleases through simpler strategies compared to heterozygous samples.

In this study, we identify a cohort of naturally occurring triple and double homozygous individuals in the Lithuanian population and isolated primary samples for prospective regenerative medicine applications. Additionally, we analyzed the frequency of HLA class I genes, specifically characterizing the *HLA-A*, *HLA-B*, and *HLA-C* haplotypes in a cohort of 3,496 individuals. The genetic makeup of the Lithuanian population is placed within a European context, influenced by pre-Neolithic Western and Scandinavian hunter–gatherer groups, Early to Middle Bronze Age steppe pastoralists, and Late Neolithic Bronze Age Europeans, while remaining largely sheltered (18). These features make the Lithuanian population closely resemble European-American (19) and British groups (20) from an immune compatibility standpoint. We compared this population to publicly available datasets of European ancestry and modeled the impact of gene editing on HLA immune matching and population coverage.

## Materials and methods

### Ethical approval

This study is part of the ethical approval 2023/6-1524-984, *Highly-immune compatible iPS cells as source for regenerative medicine and cell therapy-oriented applications*, from the Vilnius Regional Biomedical Research Ethics Committee (Lithuania) to Vilnius University, and 2023/4-1507-968, *Analysis of the distribution of Human Leukocyte Antigen (HLA; Encoding Genes - HLA) alleles and haplotypes in the group of the Lithuanian unrelated bone marrow donor registry*, to Vilnius University Hospital Santaros Klinikos. Written consent was obtained from the participants of the study.

### Study subjects

For population-based analyses of HLA frequencies, the study included 3,496 individuals from the Lithuanian unrelated bone marrow donor registry, characterized at third-field resolution for *HLA-A*, *HLA-B*, and *HLA-C*. For the isolation of dermal fibroblasts, individuals were healthy adults who provided study-specific informed consent and were selected based on their known HLA class I genotypes. Individuals aged over 55 years, those with known inherited genetic disorders, or those diagnosed with non-environmentally caused diseases were excluded from dermal biopsy collection to ensure that fibroblast samples were free from age-associated mutations or pathogenic genetic variants.

### Genotyping

HLA typing for registry donors’ peripheral blood was performed at the EFI-accredited immunogenetics laboratory at Vilnius University Hospital Santaros Klinikos (Vilnius, Lithuania) using sequencing-based typing, and at the ASHI-accredited laboratory HistoGenetics (Ossining, NY, USA) using next-generation sequencing. Exons 2 and 3 for class I HLA were covered.

### Fibroblast derivation and genotyping

Skin samples were collected using a 2–3-mm biopsy punch needle and fragmented with a sterile scalpel and needle. Fibroblasts were grown in AmnioPrime Complete Medium (cat. no. APR-B, Capricorn Scientific, Germany), supplemented with amphotericin B (cat. no. AMP-B, Capricorn Scientific, Germany), for 21 to 45 days until fibroblasts migrated from tissue sections and reached 80%–90% confluence. The medium was changed every 3 days to ensure optimal cell growth. Fibroblasts were routinely passaged with 0.25% Trypsin-EDTA at a density of 2 × 10^5^ cells/cm^2^. Genomic DNA from fibroblasts was purified using the DNeasy Blood and Tissue Kit (cat. no. 69504, Qiagen, Germany) and genotyped using the primers HLAA-P1: TCCAGGTGGACAGGTAAGGA, HLAA-P2: GTCACTGCCTGGGGTAGAAC, HLAB-P1: TGCATTCTGGGTTTCTCTACTGG, HLAB-P2: CACGCGAAACATCCCAATCA, HLAC-P1: AGGTAAGGCAAAGGGTGGGA, and HLAC-P2: AGGCCGCCTGTACTTTTCTC. Samples were Sanger sequenced using the primers HLAA-P3: ACCCTCGTCCTGCTACTCTCG, HLAB-P3: ACCCTCCTCCTGCTGCTCTG, and HLAC-P3: CGTTGGGGATTCTCCACTCC at Microsynth, Germany.

### Bioinformatics

Python and R scripts used for data analysis, along with anonymized datasets, are available through the **Supplementary Data** and or through the open-source GitHub developer platform in the repository https://github.com/Arias-Lab/superdonors.

### Quantification of HLA allele frequency in the population

The total allele count in the dataset was divided by the number of alleles (*n* = 2) times the number of individuals in this study (*n* = 3,496), all of whom had at least third-field resolution.

### Hardy–Weinberg equilibrium analyses

The observed genotypes present in the population were quantified (*n* = 3,496). The allele frequencies were determined using the sampled genotype count, and the expected genotype frequencies were calculated. The observed and expected genotype counts were compared with a χ^2^ test. The χ^2^ test is reliable for genotypes present more than five times in the population. Genotypes with a count < 5 times were filtered from the Hardy–Weinberg equilibrium (HWE) analyses. The degrees of freedom (*df*), calculated as (*n*(*n* + 1)/2) – *n*, were estimated based on the number of possible genotypes and the number of alleles identified in the sampled population for each HLA class I gene: 44 for *HLA-A*, 83 for *HLA-B*, and 45 for *HLA-C*.

### Regression analyses

Allele frequencies were extracted from the publicly available data from European-American (19) and British (20) populations and compared to the allele frequency from our study. Linear regression analyses (*y* ~ *mx* + *c*) were performed using R for pairwise comparison of allele frequencies of *HLA-A*, *HLA-B*, and *HLA-C*. Frequencies are calculated as frequency = allele count(dataset)/*n*(dataset).

### Principal component analysis

Monte Carlo population haplotypes were simulated based on the published allele frequencies of European-American and British cohort studies. Data were processed with one-hot encoding to convert allele entries per individual into 1 or 0, using the caret library in R (22). Centroids and Euclidean distances were calculated from the principal components. Distances were represented as edges and as heatmaps.

### HLA sequence analysis and sgRNA activity prediction

The sequences for all alleles at the protein, transcript, and gene levels were downloaded as FASTA files from the IPD-IMGT-HLA database version 3.58 (23) and analyzed in Python and R. Allele sequences were extracted based on the HLA alleles present in the population. Cas9 binding sites were extracted with Python and analyzed in R using CrisprScore (24). Transmembrane prediction was conducted with DeepTMHMM (25).

## Results

### Analysis of HLA class I frequencies in the Lithuanian population and identification of double and triple homozygotes

**Figure 1:**
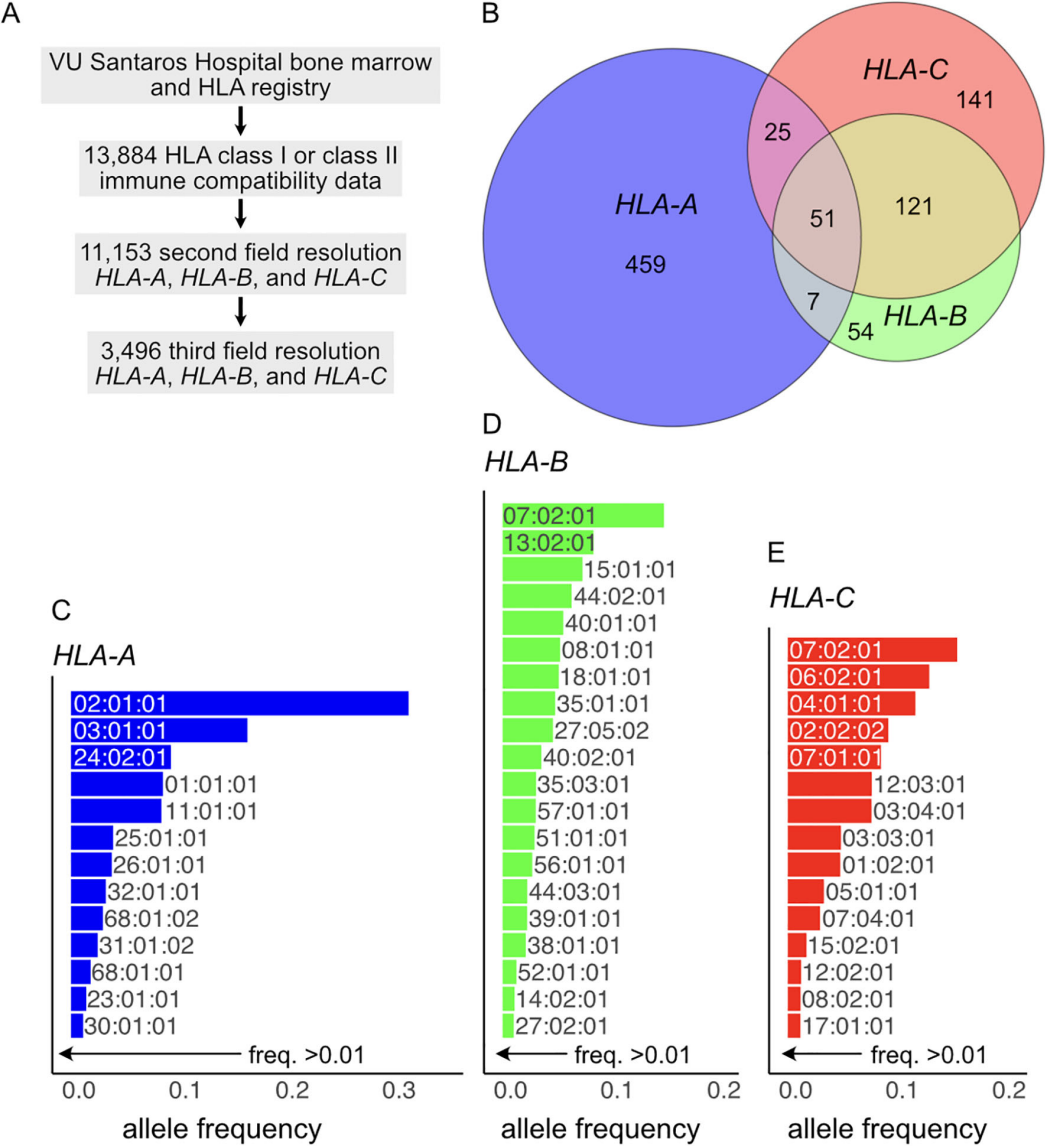
**(A)** Dataset structure from this study. **(B)** Proportional Euler diagram showing the prevalence of HLA class I homozygous individuals in the Lithuanian population, with the composition of double homozygous and triple homozygous individuals highlighted. The most common HLA alleles with a frequency above 0.01 are shown for **(C)** *HLA-A*, **(D)** *HLA-B*, and **(E)** *HLA-C*.

The Lithuanian Bone Marrow Donor Registry, located at Vilnius University Hospital Santaros Klinikos, includes 13,884 individuals, with 11,153 characterized at the second field (protein level) for *HLA-A*, *HLA-B*, and *HLA-C*. Of these, 3,496 individuals are characterized in the third field (**Figure 1A**). We found that 858 individuals are at least homozygous for one HLA class I gene. A total of 542 individuals are homozygous for the coding sequence of *HLA-A*, 233 individuals are homozygous for *HLA-B*, and 338 individuals are homozygous for *HLA-C* (**Figure 1B**). The HLA types identified and their prevalence in the population are summarized in **Figure 1** and **Supplementary Table S1**. The five most frequent *HLA-A* alleles are A*02:01:01, A*03:01:01, A*24:02:01, A*01:01:01, and A*11:01:01, which together account for 74.6% of the population (**Figure 1C**). Notably, HLA-A*02:01:01 is the most frequent HLA class I allele, representing 31.6% of the population. Similarly, the five most frequent *HLA-B* alleles are B*07:02:01, B*13:02:01, B*15:01:01, B*44:02:01, and B*40:01:01, which account for 43.2% of the population (**Figure 1D**). HLA-B*07:02:01 alone represents 15.1% of the Lithuanian population. Furthermore, the five most frequent *HLA-C* alleles are C*07:02:01, C*06:02:01, C*04:01:01, C*02:02:02, and C*07:01:01, with a cumulative frequency of 59.2% in the population (**Figure 1E**). It is important to highlight that the *HLA-B* gene exhibits the largest diversity of alleles, followed by *HLA-A* and *HLA-C* (**Supplementary Table S1**), as also observed in previous studies (19–21). Of the HLA homozygotes, a total of 153 are double homozygous (**Figure 1B**; **Supplementary Table S2**): 58 for *HLA-A* and *HLA-B*, 76 for *HLA-A* and *HLA-C*, and 172 for *HLA-B* and *HLA-C*. Remarkably, 51 individuals are triple homozygous for *HLA-A*, *HLA-B*, and *HLA-C* (**Figure 1B**; **Table 1**). Haplotype frequencies of the complete dataset (3,496 individuals) are available in the **Supplementary Data**.

**Table 1:** HLA class I triple homozygous haplotypes identified in this study (*n* = 51).

*HLA-A*	*HLA-B*	*HLA-C*	Count
A*03:01:01	B*07:02:01	C*07:02:01	15
A*02:01:01	B*13:02:01	C*06:02:01	10
A*01:01:01	B*08:01:01	C*07:01:01	6
A*02:01:01	B*07:02:01	C*07:02:01	4
A*02:01:01	B*40:01:01	C*03:04:01	3
A*02:01:01	B*57:01:01	C*06:02:01	2
A*03:01:01	B*56:01:01	C*01:02:01	2
A*25:01:01	B*18:01:01	C*12:03:01	2
A*02:01:01	B*15:01:01	C*03:04:01	1
A*02:01:01	B*27:05:02	C*02:02:02	1
A*25:01:01	B*35:01:01	C*04:01:01	1
A*26:01:01	B*38:01:01	C*12:03:01	1
A*31:01:02	B*51:01:01	C*05:01:01	1
A*68:01:01	B*40:01:01	C*03:04:01	1
A*68:01:02	B*44:02:01	C*07:04:01	1

### Comparisons of HLA class I allele composition between populations

**Figure 2:**
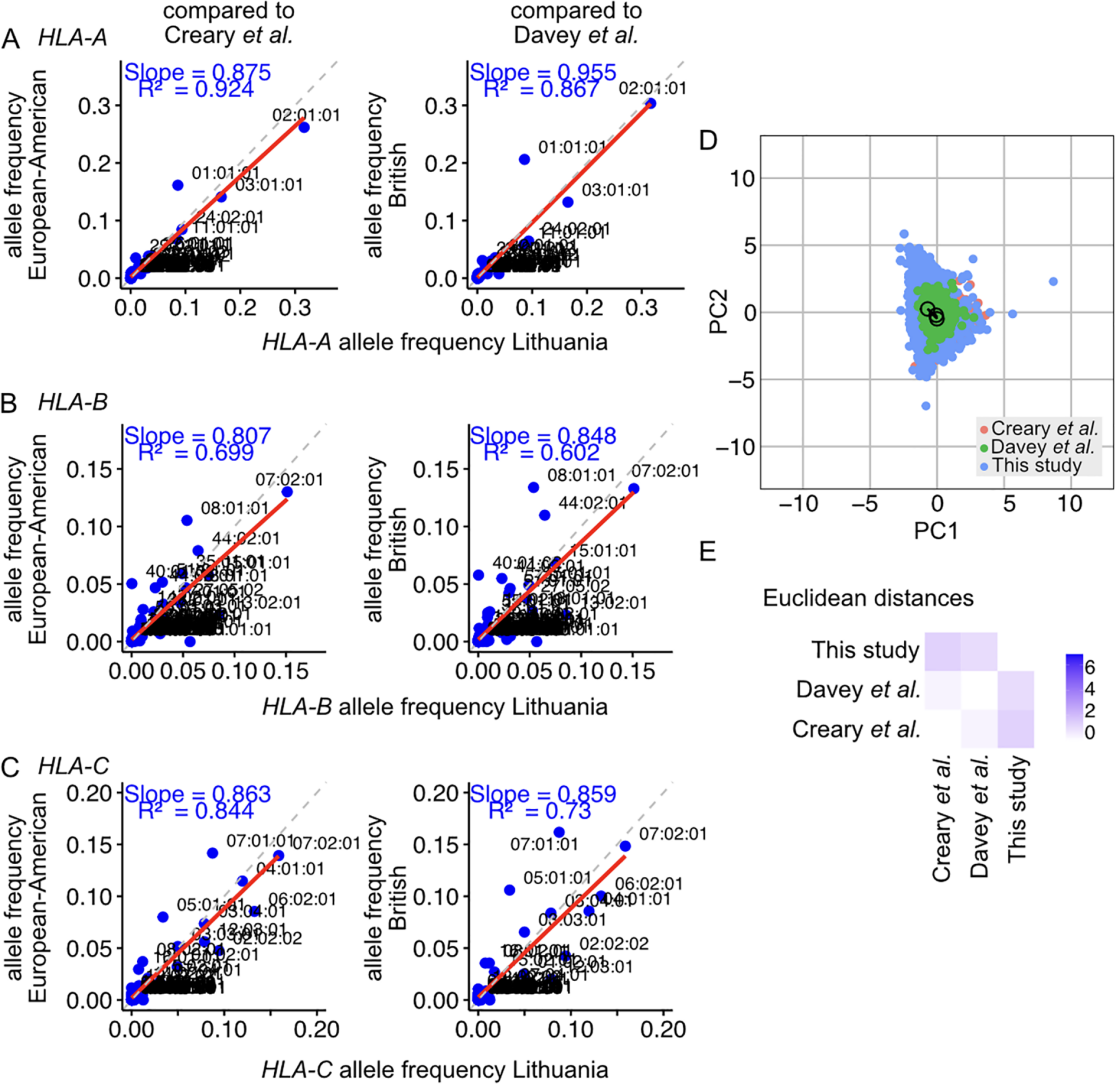
Comparison of the HLA allele frequencies identified in the Lithuanian population with those reported in studies of the European-American and the British population for **(A)** the *HLA-A* transcript, **(B)** the *HLA-B* transcript, and **(C)** the *HLA-C* transcript. Reference lines with slope *n* = 1 are represented as dashed grey lines. The linear regressions of frequencies on the scatter plots are represented with a solid red line, with the *R* ^2^ of the linear model and the slope indicated. **(D)** Principal component analysis of the HLA class I distribution in the Lithuanian population (this study) and other populations, including European-American and British cohorts. The centroid of each population is marked with a circle. The Euclidean distances between the centroids were calculated, and the edges are plotted with solid lines. **(E)** Euclidean distance heatmap between the studied populations. Blue corresponds to greater Euclidean distances in the principal component space.

Comparisons of the Lithuanian Class I HLA frequencies with those reported for the European-American and British populations using linear regression models show strong correlations between the three cohorts (**Figures 2A–C**). The linear regression analyses yielded an average slope of 0.914 for *HLA-A*, 0.827 for *HLA-B*, and 0.860 for *HLA-C*. This indicates the populations closely resemble each other in the composition and prevalence of allele variants. Principal component analysis (PCA) was performed on the genotypes of the Lithuanian population and on genotypes reconstructed from published datasets using Monte Carlo analysis based on reported allele frequencies. The results showed that the Lithuanian population clustered in close proximity to the compared populations (**Figure 2D**). The Euclidean distances between the centroids of the populations were quantified and represented in the PCA and as a heatmap (**Figure 2E**). The distance metrics indicate that the centroid of the Lithuanian population is proximal to the European-American and British populations, with distances of 1.00 and 0.79 relative units, respectively. The British and European-American populations closely resemble each other, with a Euclidean distance of 0.27 relative units. Hardy–Weinberg equilibrium analyses show that some genotypes, including the 10 most frequent allele types, occur at higher frequencies than expected (**Supplementary Data**; **Supplementary Figure S1**).

### Compatibility of HLA class I in the Lithuanian and other European populations

**Figure 3:**
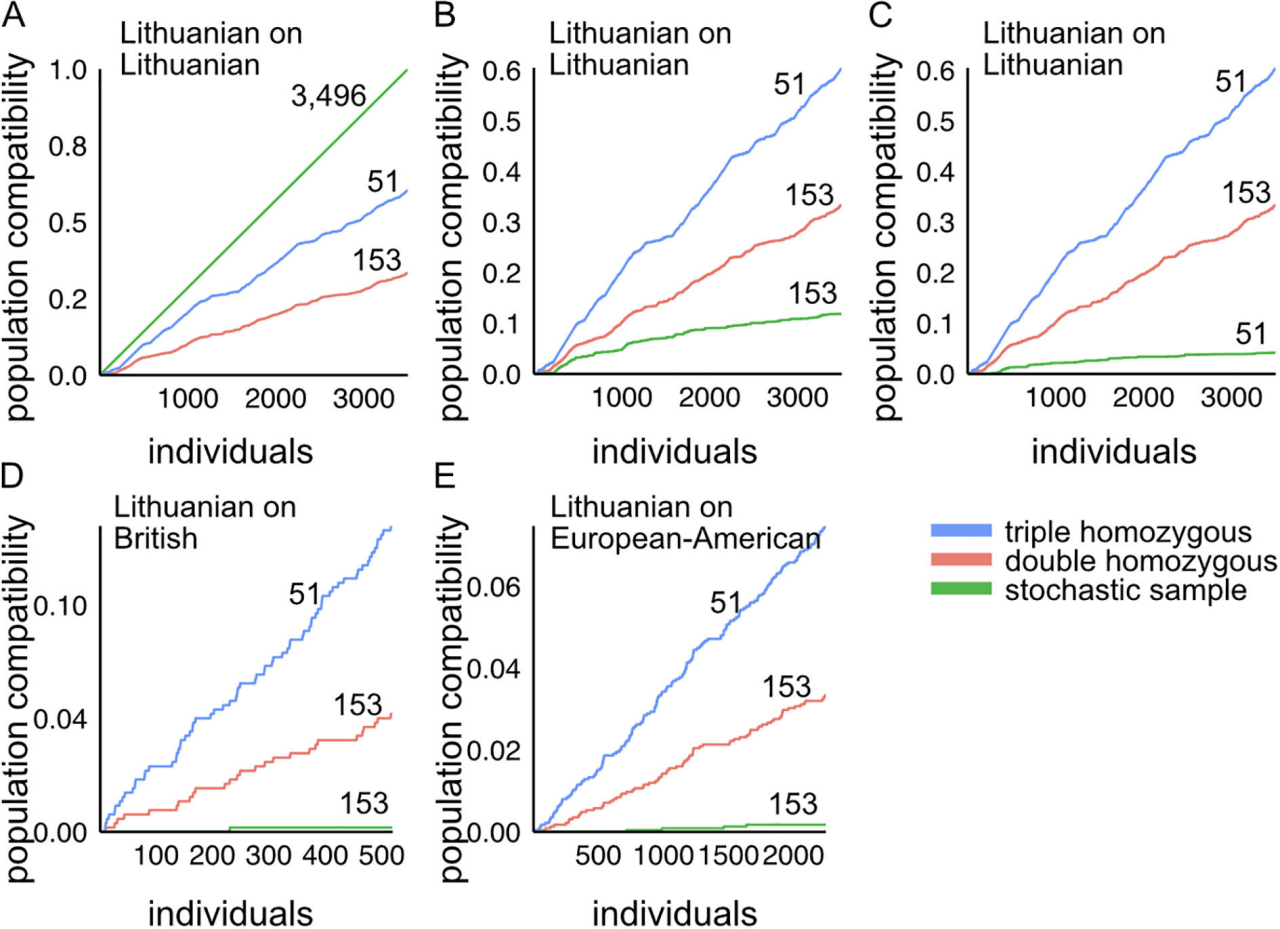
Population compatibility of *HLA-A*, *HLA-B*, and *HLA-C* genotypes in Lithuanian samples with Lithuanian and other European populations. Immune compatibility of triple homozygous (51 individuals), double homozygous (153 individuals), and **(A)** all samples from the cohort of 3,496 individuals in this study, **(B)** stochastically selected samples of 153 individuals, and **(C)** stochastically selected samples of 51 individuals. Immune compatibility of HLA class I genes, *HLA-A*, *HLA-B*, and *HLA-C*, in Lithuanian samples with **(D)** British datasets and **(E)** European-American datasets. Triple homozygous individuals are indicated in blue, double homozygous individuals in red, and stochastically selected subsamples in green.

We stochastically arranged the 3,496 donors and interrogated whether the subset of *HLA-A*, *HLA-B*, and *HLA-C* triple homozygous (51 samples) and double homozygous (153 samples) individuals were compatible with the 3,496 patients (**Figure 3**). We found that our cohort of triple homozygous individuals matches 60.46% of the Lithuanian population (**Figure 3A**). Likewise, the double homozygous cohort matches 33.32% of the Lithuanian population. In comparison, a randomly selected subset of 153 or 51 samples from the dataset could match only 11.84% (**Figure 3B**) and 4.1% (**Figure 3C**) of the Lithuanian population, respectively. We then evaluated the matching provided by our triple homozygous and double homozygous cohorts to the European-American and British populations. We assessed their immune compatibility with Monte Carlo datasets reconstructed from allele frequencies reported for European-American and British individuals. Remarkably, we found that the 51 triple homozygous samples of our cohort match 13.4% of the British population, while the double homozygous cohort matches 5.2% (**Figure 3D**). Additionally, we found that triple homozygous samples match 7.4% of the European-American population, and double homozygous samples match 3.3% (**Figure 3E**).

### Cas9 activity prediction on HLA class I alleles of the Lithuanian population

**Figure 4:**
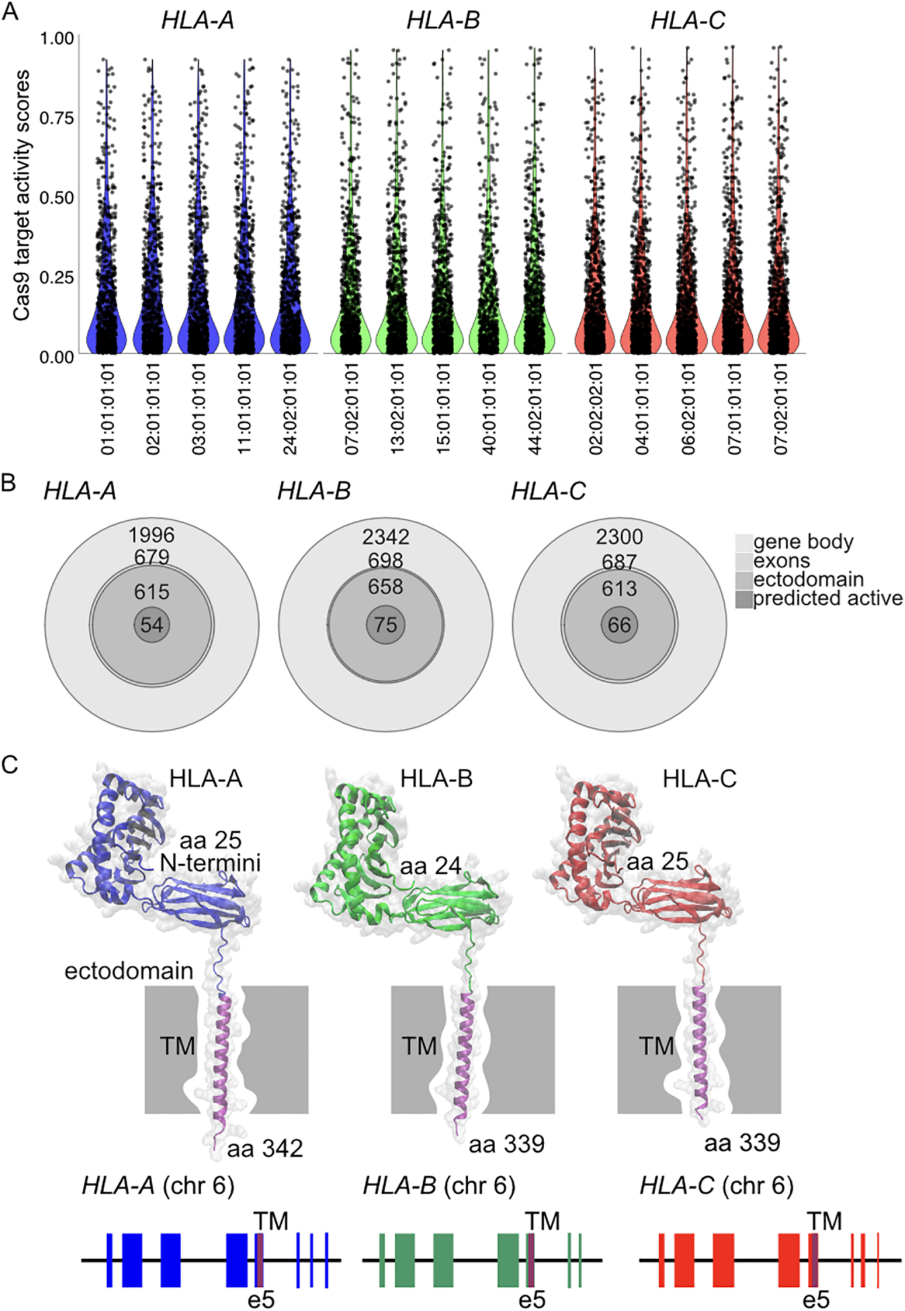
Predicted guide RNA sequence activity for the five most frequent alleles in the Lithuanian population for **(A)** *HLA-A*, *HLA-B*, and *HLA-C*. **(B)** Nested distribution of guide RNAs on the gene body, exons, and ectodomain, as well as those with predicted high activity for *HLA-A*, *HLA-B*, and *HLA-C*. **(C)** Protein structure models and gene structures for HLA-A, HLA-B, and HLA-C. Protein structures are depicted as mature forms, excluding the signal peptide and the highly flexible endodomain. Gene structures highlight the matching ectodomain and transmembrane (TM) region.

We extracted the Cas9-binding site sequences from the HLA alleles present in the Lithuanian population. First, we focused on the analysis of target regions encompassing the gene body, from the 5′UTR to the 3′UTR. We found 1,996 unique target sites in *HLA-A* alleles, 2,342 unique target sites in *HLA-B*, and 2,300 unique target sites in *HLA-C*. We calculated the activity prediction score based on the rule set 1 of nuclease catalytic activity (26). We found that, as in non-hyper polymorphic genes, the activity scores of all HLA alleles are centered in the inactive Q4 quadrant. We show this distribution for the five most frequent alleles of *HLA-A*, *HLA-B*, and *HLA-C* (**Figure 4A**). The potential of HLA gene knockout to modulate immune compatibility is well accepted in the literature. Although pairs of guide RNAs can be used in conjunction to create exon-spanning knockouts, we focused on guide RNAs in exon regions. From the guide RNAs present in the gene body, we found 679 unique target sites in the *HLA-A* exons of Lithuanian alleles, 698 in *HLA-B*, and 687 in *HLA-C* (**Figure 4B**). Since *HLA-A*, *HLA-B*, and *HLA-C* are class I single-span transmembrane proteins (**Figure 4C**), only guide RNAs targeting the ectodomain have the capacity to create knockouts that eliminate plasma membrane expression of HLA genes. We predicted the transmembrane spanning region (25) of the allele sequences and focused on guide RNAs directed to the N-terminus, upstream of the predicted transmembrane domain. We found there are 615 unique target sites in Lithuanian alleles on *HLA-A* ectodomains, 658 on *HLA-B*, and 613 on *HLA-C* (**Figure 4B**). Of those useful for ectodomain targeting, a fraction have predicted activity scores greater than 0.5. These include 54 for *HLA-A*, 75 for *HLA-B*, and 66 for *HLA-C* (**Figure 4B**).

### Modeling the impact of HLA class I engineering on the immune compatibility of triple homozygous and double homozygous donor samples

**Figure 5:**
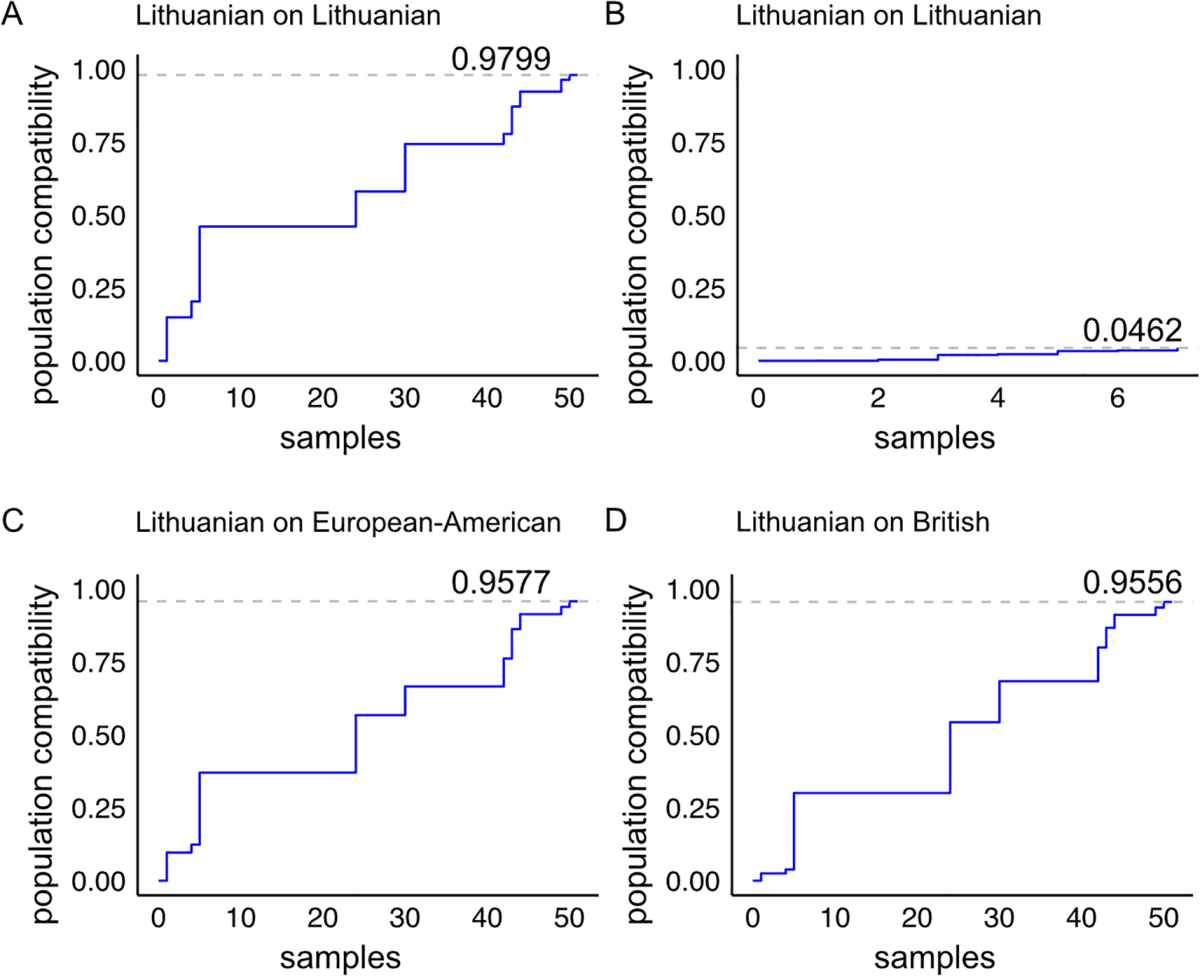
Population compatibility model of *HLA-A* and *HLA-B* double knockout samples from our cohort with the Lithuanian population and with populations of European-American and British ancestry. **(A)** Immune compatibility of the 51 triple homozygous individuals in an *HLA-A* and *HLA-B* double knockout model, and **(B)** the seven double homozygous individuals in an *HLA-A* and *HLA-B* double knockout model when matched to the Lithuanian population. The cohort from **(A)** matched with **(C)** the European-American dataset and **(D)** the British dataset.

Naturally occurring triple and double homozygous samples are particularly useful for gene engineering approaches as they allow bi-allelic targeting with a single programmable nuclease in a one-step intervention. Next, we modelled the impact of *HLA-A* and *HLA-B* knockouts on the immune compatibility of the double and triple homozygous samples when matching them to the Lithuanian population and other European datasets (**Figure 5**). We included all 51 triple homozygous individuals from our cohort (**Figure 5A**). From the 153 double homozygous individuals identified, we focused on those that are *HLA-A* and *HLA-B* double homozygous, comprising seven individuals (**Figure 5B**). The 51 triple homozygous samples, when in an *HLA-C*-retained (*HLA-A* and *HLA-B* double knockout) configuration, match a maximum of 0.9799 of the Lithuanian population (**Figure 5A**). These 51 samples achieve a match of 0.9577 in the European-American population (**Figure 5C**) and 0.9556 in the British population (**Figure 5D**).

### Sampling of *HLA-A*, *HLA-B*, and *HLA-C* triple homozygous individuals from the Lithuanian population

**Figure 6:**
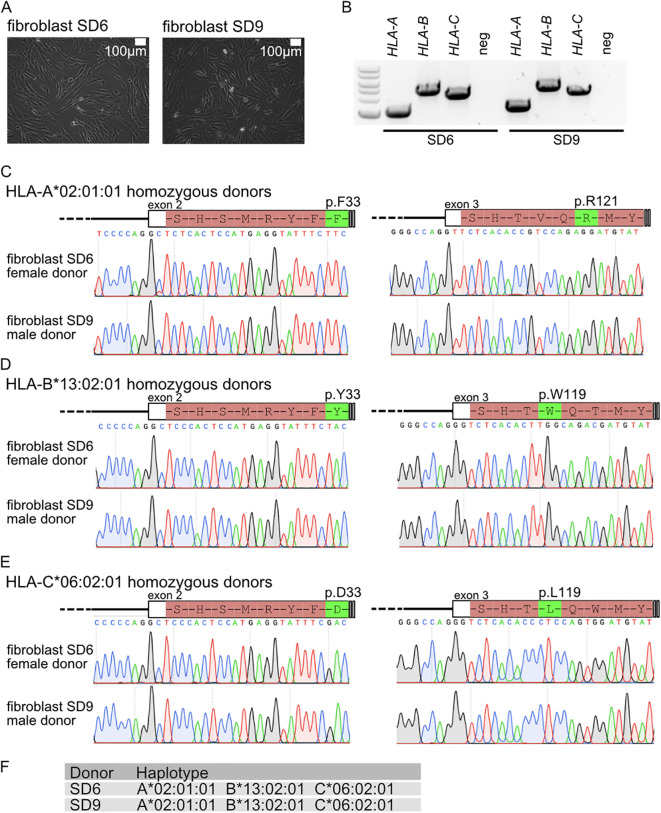
**(A)** Fibroblast cultures from *HLA-A*, *HLA-B*, and *HLA-C* triple homozygous donors. **(B)** Genotyping PCR for *HLA-A*, *HLA-B*, and *HLA-C*. Sanger sequencing analysis for **(C)** *HLA-A*, **(D)** *HLA-B*, and **(E)** *HLA-C*. **(F)** Haplotype for donor patient and linked fibroblasts.

Since the triple homozygote individuals identified in this study are immune-compatible with a large fraction of the Lithuanian and other European populations, we sampled these volunteers. The collected dermal fibroblast samples were used to establish biobank stocks and cultures. Primary fibroblast cultures were robustly established for 15 triple homozygotes (**Figure 6A**; **Supplementary Table S3**). PCR products of exons 2 and 3 (**Figure 6B**) display single bands, and Sanger sequencing yields clear chromatograms, both of which are characteristic of homozygous samples (**Figures 6C–E**). Sanger sequencing of exons 2 and 3, which code for the ectodomains of *HLA-A*, *HLA-B*, and *HLA-C*, revealed characteristic residues for each allele. Characteristic amino acids p.F33 and p.R121 were confirmed for HLA-A*02:01:01 (**Figure 6C**), p.Y33 and p.W119 for HLA-B*13:02:01 (**Figure 6D**), and p.D33 and p.L119 for HLA-C*06:02:01 (**Figure 6E**). These findings were consistent for both XY (donor SD9) and XX (donor SD6) individuals with the homozygous haplotype *HLA-A*02:01:01-*HLA-B*13:02:01-*HLA-C*06:02:01 (**Figure 6F**).

## Discussion

Our study on allele and haplotype frequencies of the *HLA-A*, *HLA-B*, and *HLA-C* genes in the Lithuanian population elucidates immune compatibility structure in relation to other European populations. Comparative analyses confirmed high similarity in HLA class I genes between the Lithuanian population and populations of European-American and British ancestry. The most frequent alleles described in the British (20) and European-American populations (19) are also the most frequent in the Lithuanian population, with frequencies of 31.6% (A*02:01:01), 5.3% (B*08:01:01), 15.1% (B*07:02:01), and 8.7% (C*07:01:01). Linear regression analysis using publicly available data corroborated these observations. PCA and Euclidean distance calculations further confirmed the proximity in immune compatibility among Lithuanian, European-American, and British populations. HWE analysis revealed deviations in a subset of alleles, suggesting partial genetic isolation or selective pressure. These findings align with previous studies indicating low levels of admixture and a significant component of pre-Neolithic hunter–gatherer ancestry in the Lithuanian group (18).

The majority of individuals in our registry (*n* = 11,153) were characterized at second-field resolution for *HLA-A*, *HLA-B*, and *HLA-C*, while a subset (*n* = 3,496) underwent third-field resolution analysis. This divergence reflects technological advancements in clinical registries, with long-read sequencing platforms now enabling fourth-field resolution (27, 28). Although our analyses do not encompass HLA class II, it is well established that its expression occurs in specialized immune cell lineages, whereas HLA class I primarily regulates nonimmune and immune cell compatibility (29, 30). The exclusive focus on HLA class I represents a potential limitation of this study, especially considering the importance of HLA class II matching in immunotherapeutic applications. Remarkably, we found a subset of 51 triple homozygous individuals for *HLA-A*, *HLA-B*, and *HLA-C*, and a subset of 153 double homozygous individuals. The proportion of triple-homozygous individuals exceeded stochastic expectations based on measured allele frequencies (2.99 ± 1.76), suggesting underlying population structures, as indicated by HWE analysis.

Due to the significant immune compatibility provided by *HLA-A*, *HLA-B*, and *HLA-C* triple homozygous individuals (31, 32), the term naturally occurring *superdonors* has been proposed previously (33). Our study identified 51 naturally occurring *superdonors* who exhibit HLA class I immune matching with 60.46% of the Lithuanian population, 13.4% of the British population, and 7.4% of the European-American population. These populations exhibit similar individual allele frequencies, yet their reduced HLA class I immune matching is likely due to differences in haplotype composition. It is important to highlight that using triple homozygous samples for cell line development, particularly human iPS cells, results in derivatives with wider immune compatibility than heterozygous counterparts for nonimmune cell identities. Genetic engineering with programmable nucleases in such samples benefits from simpler strategies because of the homozygosity status of the starting material. In turn, engineered products are expected to attain broader immune compatibility than natural counterparts.

Several international initiatives focus on iPS cell development from haplo-selected individuals, including programs in Japan (34), Australia (33), South Korea (35, 36), Spain (37), Germany (38), Lithuania, and Saudi Arabia (39). We modeled the impact of *HLA-C-retained* gene-editing intervention on the 51 naturally occurring *superdonors* and found that their immune compatibility could be enhanced to match 97.9% of the Lithuanian population, 95.7% of the European-American population, and 95.5% of the British population. Conversely, the immune compatibility provided by the *HLA-A* and *HLA-B* double-homozygous individuals was limited due to the retained diversity within the heterozygous *HLA-C* allele.

Here, we propose the term *synthetic superdonor* for those cell lines derived from naturally occurring *superdonors* that, through gene editing, acquire broader immune compatibility. Analysis of gene-editing availability for *HLA-A*, *HLA-B*, and *HLA-C* highlights the importance of protein topology, knockout strategy design, and nuclease target site activity in achieving *synthetic superdonor* stocks.The HLA-A, HLA-B, and HLA-C proteins are of the type I transmembrane class; hence, targeting the N-terminus ectodomain slightly constrains the number of available Cas9-binding sites. Our analyses demonstrate that the largest impact on knockout availability is the nuclease activity score; therefore, gene-editing tools that enhance nuclease activity are likely to have a positive impact on *synthetic superdonor* creation in the future. Likewise, our analyses indicate that naturally occurring *superdonor* and *synthetic superdonor* cell sources would positively impact immune matching for rare haplotypes. Both naturally occurring and *synthetic superdonors* are a remarkable source for the creation of iPS cells and derivative advanced therapeutic medicinal products (ATMPs).

## Data Availability

The datasets presented in this study can be found in **Supplementary Materials** and online repositories listed in article (Materials and Methods section) **Supplementary Materials**.

## Supplementary material

Supplementary Figure 1 Hardy-Weinberg equilibrium (HWE) analysis for **(A)** *HLA-A*, *HLA-B* and *HLA-C* genotypes. The heatmaps represent the ratio between observed genotype frequencies and expected genotype frequencies. Only the genotypes were *X* ^2^ value exceeds the *X* ^2^-threshold, indicating a HWE deviation are displayed. The subset of genotypes with a frequency higher than 0.01 are represented in heatmaps.

Supplementary Figure 2 Cumulative-coverage of HLA class I immune matching in the Lithuanian population. Individuals in our dataset were sampled, and their cumulative-coverage in the population was calculated with 100 sampling iterations for **(A)** 1,000 individuals, and **(B)** 329 individuals, which is the estimated sample size to reach a 1-time cumulative-coverage. **(C)** Sampling of 1,000 and **(D)** 329 individuals excluding the possibility of autologous donation. **(E)** Cumulative-coverage for the individuals found to be double homozygous (red) and triple homozygous (blue) for *HLA-A*, *HLA-B* and *HLA-C*. Comparison with 1,000 randomly sampled individuals in 100 iterations (grey). The average cumulative coverage of all iterations is shown in black.

Supplementary Table 1 HLA class I allele frequencies observed in the Lithuanian population (n = 3,496).

Supplementary Table 2 HLA class I double homozygous haplotypes identified in this study (n = 153).

Supplementary Table 3
*HLA-A*, *HLA-B* and *HLA-C* triple homozygous fibroblasts derived in this study.
