# Supplementary material for: Identification of HLA-A, HLA-B, and HLA-C triple homozygous and double homozygous donors: a path toward synthetic superdonor advanced therapeutic medicinal products
Source: Front Immunol. 2025 Sep 16;16:1626787. doi: 10.3389/fimmu.2025.1626787 (PMC12479312; doi:10.3389/fimmu.2025.1626787)
Supplement: Supplementary Figure 1 — Hardy-Weinberg equilibrium (HWE) analysis for (A) HLA-A, HLA-B and HLA-C genotypes. The heatmaps represent the ratio between observed genotype frequencies and expected genotype frequencies. Only the genotypes were X 2 value exceeds the X 2-threshold, indicating a HWE deviation are displayed. The subset of genotypes with a frequency higher than 0.01 are represented in heatmaps. [file DataSheet1.docx]

**Supplementary information**

**Identification of HLA-A, HLA-B and HLA-C triple homozygous and double homozygous donors: a path towards synthetic superdonor Advanced Therapeutic Medicinal Products**

Daniel Naumovas 1 2 3, Barbara Rojas-Araya 1, Catalina M. Polanco 1, Victor Andrade 4 5, Rita Čekauskienė 3, Beatričė Valatkaitė-Rakštienė 3, Inga Laurinaitytė 2, Artūras Jakubauskas 3, Mindaugas Stoškus 3, Laimonas Griškevičius 3, Ivan Nalvarte 6, Jose Inzunza 7 8, Daiva Baltriukienė 9, Jonathan Arias 1 7 8 *

1 Laboratory of Nuclease Enabled Cell Therapies, Vilnius University Life Science Center-EMBL Partnership Institute for Gene Editing Technologies, 2 Vilnius Santaros Klinikos Biobank, Vilnius University Hospital Santaros Klinikos, Vilnius, Lithuania, 3 Department of Molecular Medicine; Hematology, Oncology and Transfusion Medicine Center, Vilnius University Hospital Santaros Klinikos, Vilnius, Lithuania, 4 Division of Neurogenetics and Molecular Psychiatry, Department of Psychiatry and Psychotherapy, Medical Faculty, University of Cologne, Cologne, Germany, 5 Department of Cognitive Disorders and Old Age Psychiatry, University Hospital Bonn, Bonn, Germany, 6 Karolinska Institutet, Department of Neurobiology, Care Sciences and Society, BioClinicum, Solna, Sweden, 7 Karolinska Institutet Stem Cell Organoid (KISCO) facility, Department of Laboratory Medicine, Huddinge, Sweden, 8 Karolinska Institutet, Department of Laboratory Medicine, Huddinge, Sweden, 9 Department of Biological Models, Institute of Biochemistry, Life Sciences Center, Vilnius University, Vilnius, Lithuania

* correspondence: Jonathan Arias jonathan.arias@gmc.vu.lt

**Population analysis for HLA class I composition.** We conducted a Hardy-Weinberg equilibrium analysis to determine whether there were deviations in the genotype frequencies of the Lithuanian HLA class I allele pool compared to the expected frequencies under Hardy-Weinberg conditions for each allele. We found 44 alleles for *HLA-A*, 83 alleles for *HLA-B*, and 45 alleles for *HLA-C* within the 3,496 individuals analyzed (Supplementary table 1). This diversity of allele types results in a maximum of 990 genotypes for *HLA-A*, 3,486 for *HLA-B*, and 1,035 for *HLA-C*. When excluding the rare variants, defined as those with a frequency of less than 0.01 in the population, we found that 22 genotypes deviate from Hardy-Weinberg equilibrium for *HLA-A*, 16 deviate for *HLA-B*, and 30 deviate for *HLA-C* (Supplementary figure 1). The highest-ranked *HLA-A* genotype that is present more frequently than expected is HLA-A*03:01:01-25:01:01, which combines two of the top six most abundant allele types (Supplementary figure 1). Likewise, HLA-B*13:02:01-27:05:02, HLA-B*08:01:01-15:01:01, and HLA-B*15:01:01-44:02:01, which are combinations of the top ten most frequent allele types, are present at higher frequencies than expected under Hardy-Weinberg equilibrium conditions (Supplementary figure 1). Furthermore, a larger group of *HLA-C* genotypes deviate from equilibrium, including genotypes of the most frequent allele types HLA-C*07:01:01-12:03:01, HLA-C*01:02:01-04:01:01, HLA-C*02:02:02-06:02:01, HLA-C*04:01:01-06:02:01, HLA-C*03:04:01-12:03:01, HLA-C*01:02:01-06:02:01, and HLA-C*07:02:01-07:02:01 (Supplementary figure 1). In fact, HLA-C*07:02:01 is the most frequent allele type, and its homozygous combination is enriched more than expected based on Hardy-Weinberg equilibrium conditions.

**HLA class I immune compatibility cumulative-coverage based on stochastic sampling, double homozygous or triple homozygous sampling.** The cumulative-coverage accounts for the extent of redundancy in the population, which allows for matching even when a single sample may not be accessible for donation. Using the third-field HLA information for *HLA-A*, *HLA-B* and *HLA-C* from 3,496 individuals from this study, we conducted sampling simulations and calculated the cumulative coverage of 1,000 randomly selected individuals from the Lithuanian population. Using 100 simulations, we found that, on average, 1,000 randomly selected samples achieve a cumulative-coverage of 3.1 ± 0.4 times the population (Supplementary figure 2A). We found that approximately 329 individuals provide an average cumulative-coverage of 0.99 ± 0.2 of the populations (Supplementary figure 2B). We then excluded the possibility of autologous donation from the population’s cumulative-coverage analysis. When randomly selecting 1,000 individuals in 100 sampling iterations, a lower cumulative-coverage was achieved, with an average of 2.7 ± 0.4 times the population (Supplementary figure 2C). Sampling 329 individuals, excluding the possibility of autologous donation, results in a cumulative-coverage of only 0.9 ± 0.2 times the population (Supplementary figure 2D). Remarkably, when evaluating the cumulative-coverage of double or triple homozygous for *HLA-A*, *HLA-B* and *HLA-C*, a higher cumulative-coverage is achieved with a smaller set of samples. The 153 double homozygous samples achieve a cumulative-coverage of 2.2 times the population, and the 51 triple homozygous samples yield a cumulative-coverage of 4.9 times the population (Supplementary figure 2E). A side-by-side comparison of the double and triple homozygous cumulative-coverage with stochastic sampling of 1,000 individuals highlights the impact of homozygosity on HLA class I immune compatibility to the population (Supplementary figure 2E).

**Supplementary figures:**

**Supplementary figure 1**


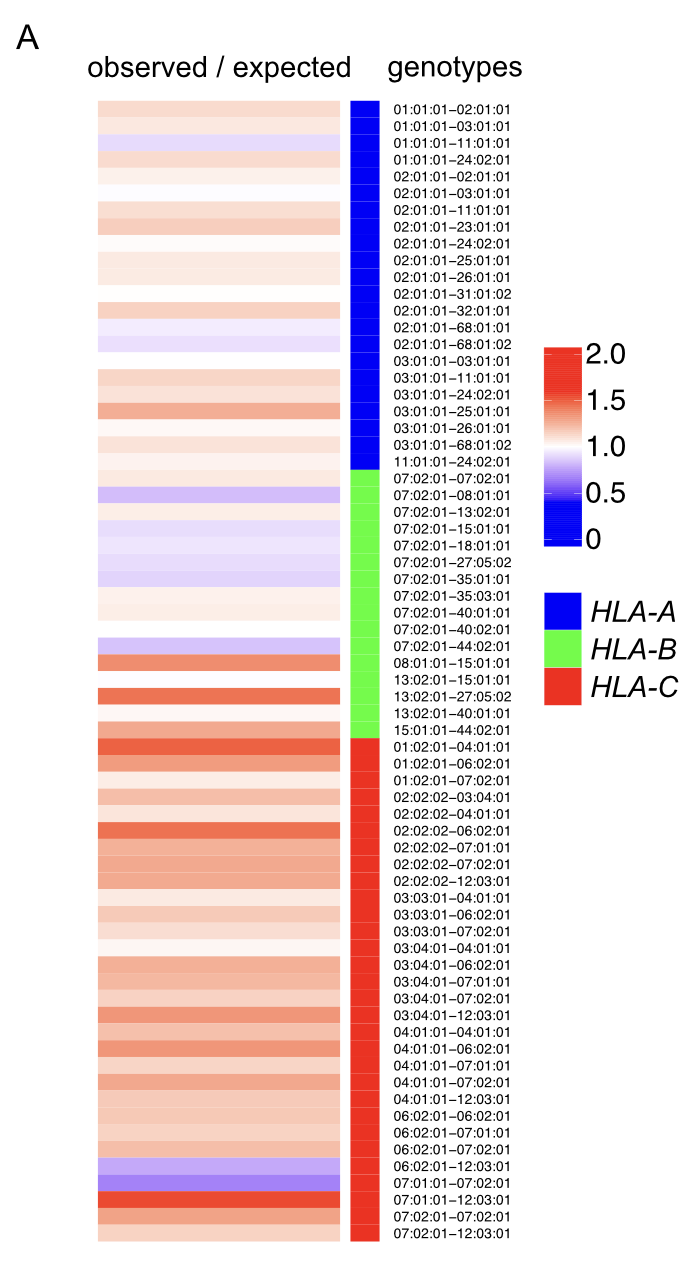


**Supplementary figure 2**


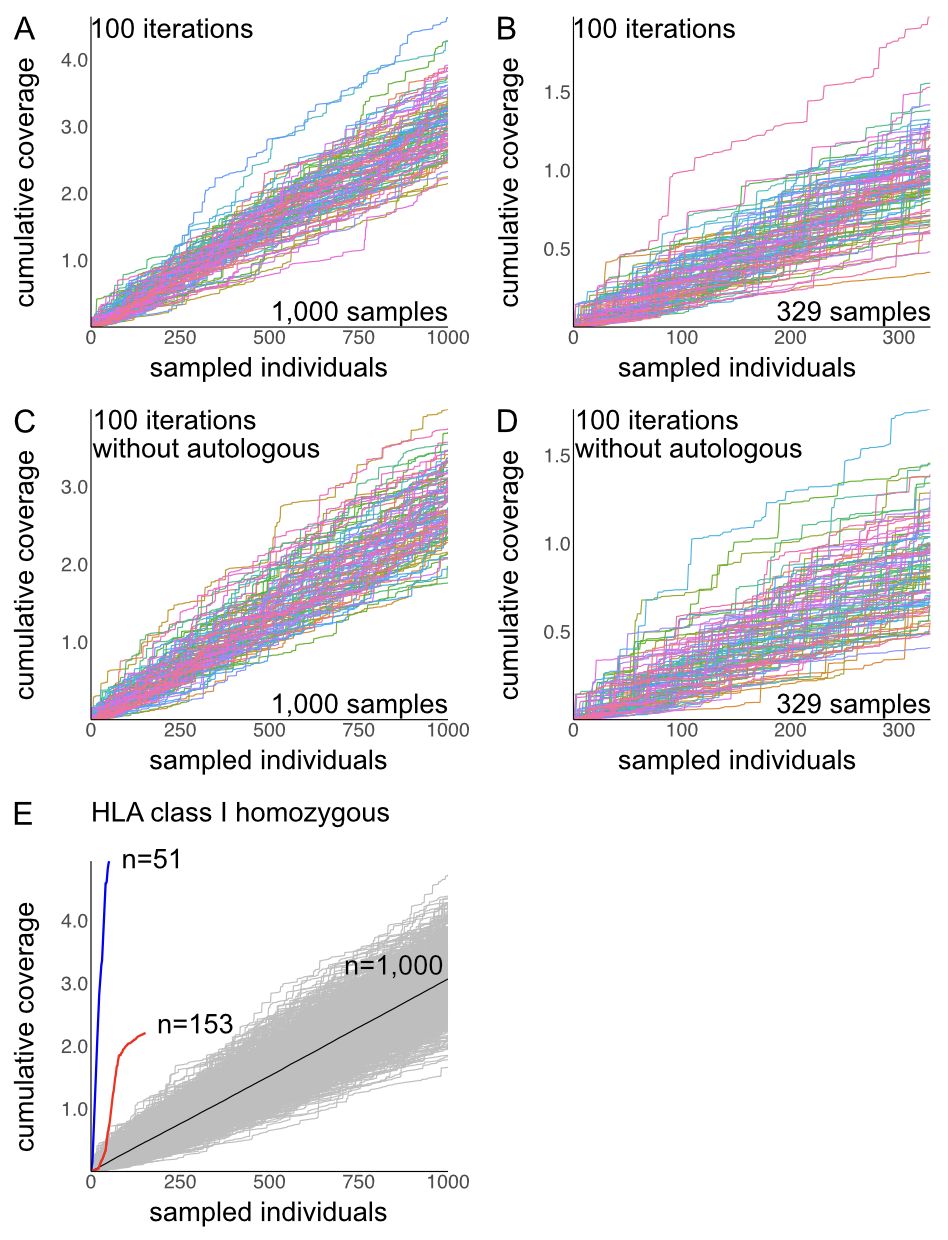


**Supplementary figure legends**

**Supplementary figure 1.** Hardy-Weinberg equilibrium (HWE) analysis for A. *HLA-A*, *HLA-B* and *HLA-C* genotypes. The heatmaps represent the ratio between observed genotype frequencies and expected genotype frequencies. Only the genotypes were *X*^2^ value exceeds the*X*^2^-threshold, indicating a HWE deviation are displayed. The subset of genotypes with a frequency higher than 0.01 are represented in heatmaps.

**Supplementary figure 2.** Cumulative-coverage of HLA class I immune matching in the Lithuanian population. Individuals in our dataset were sampled, and their cumulative-coverage in the population was calculated with 100 sampling iterations for A. 1,000 individuals, and B. 329 individuals, which is the estimated sample size to reach a 1-time cumulative-coverage. C. Sampling of 1,000 and D. 329 individuals excluding the possibility of autologous donation. E. Cumulative-coverage for the individuals found to be double homozygous (red) and triple homozygous (blue) for *HLA-A*, *HLA-B* and *HLA-C*. Comparison with 1,000 randomly sampled individuals in 100 iterations (grey). The average cumulative coverage of all iterations is shown in black.

**Supplementary tables:**

**Supplementary table 1.** HLA class I allele frequencies observed in the Lithuanian population (n = 3,496)

| HLA-A | count | freq | HLA-B | count | freq | HLA-C | count | freq |
| --- | --- | --- | --- | --- | --- | --- | --- | --- |
| 02:01:01 | 2212 | 0.3164 | 07:02:01 | 1057 | 0.1512 | 07:02:01 | 1110 | 0.1588 |
| 03:01:01 | 1156 | 0.1653 | 13:02:01 | 595 | 0.0851 | 06:02:01 | 927 | 0.1326 |
| 24:02:01 | 655 | 0.0937 | 15:01:01 | 523 | 0.0748 | 04:01:01 | 836 | 0.1196 |
| 01:01:01 | 602 | 0.0861 | 44:02:01 | 452 | 0.0646 | 02:02:02 | 659 | 0.0943 |
| 11:01:01 | 592 | 0.0847 | 40:01:01 | 397 | 0.0568 | 07:01:01 | 611 | 0.0874 |
| 25:01:01 | 276 | 0.0395 | 08:01:01 | 376 | 0.0538 | 12:03:01 | 551 | 0.0788 |
| 26:01:01 | 267 | 0.0382 | 18:01:01 | 368 | 0.0526 | 03:04:01 | 549 | 0.0785 |
| 32:01:01 | 228 | 0.0326 | 35:01:01 | 344 | 0.0492 | 03:03:01 | 349 | 0.0499 |
| 68:01:02 | 209 | 0.0299 | 27:05:02 | 329 | 0.0471 | 01:02:01 | 344 | 0.0492 |
| 31:01:02 | 175 | 0.0250 | 40:02:01 | 254 | 0.0363 | 05:01:01 | 237 | 0.0339 |
| 68:01:01 | 129 | 0.0184 | 35:03:01 | 219 | 0.0313 | 07:04:01 | 212 | 0.0303 |
| 23:01:01 | 100 | 0.0143 | 57:01:01 | 217 | 0.0310 | 15:02:01 | 123 | 0.0176 |
| 30:01:01 | 80 | 0.0114 | 51:01:01 | 209 | 0.0299 | 12:02:01 | 88 | 0.0126 |
| 29:02:01 | 64 | 0.0092 | 56:01:01 | 194 | 0.0277 | 08:02:01 | 84 | 0.0120 |
| 33:01:01 | 42 | 0.0060 | 44:03:01 | 161 | 0.0230 | 17:01:01 | 83 | 0.0119 |
| 66:01:01 | 29 | 0.0041 | 39:01:01 | 160 | 0.0229 | 14:02:01 | 56 | 0.0080 |
| 02:05:01 | 24 | 0.0034 | 38:01:01 | 151 | 0.0216 | 16:01:01 | 53 | 0.0076 |
| 68:02:01 | 24 | 0.0034 | 52:01:01 | 90 | 0.0129 | 03:02:01 | 21 | 0.0030 |
| 29:01:01 | 21 | 0.0030 | 14:02:01 | 78 | 0.0112 | 02:02:01 | 18 | 0.0026 |
| 33:03:01 | 21 | 0.0030 | 27:02:01 | 73 | 0.0104 | 16:02:01 | 12 | 0.0017 |
| 03:02:01 | 15 | 0.0021 | 49:01:01 | 65 | 0.0093 | 07:68 | 11 | 0.0016 |
| 02:06:01 | 9 | 0.0013 | 55:01:01 | 64 | 0.0092 | 15:05:01 | 10 | 0.0014 |
| 31:01:01 | 9 | 0.0013 | 37:01:01 | 63 | 0.0090 | 16:04:01 | 9 | 0.0013 |
| 30:04:01 | 7 | 0.0010 | 27:05:03 | 56 | 0.0080 | 07:02:08 | 4 | 0.0006 |
| 02:07:01 | 6 | 0.0009 | 41:02:01 | 56 | 0.0080 | 15:04:01 | 4 | 0.0006 |
| 02:35:01 | 6 | 0.0009 | 44:05:01 | 56 | 0.0080 | 08:01:01 | 3 | 0.0004 |
| 30:02:01 | 5 | 0.0007 | 35:02:01 | 52 | 0.0074 | 17:03:01 | 3 | 0.0004 |
| 03:22:01 | 4 | 0.0006 | 41:01:01 | 39 | 0.0056 | 18:01:01 | 3 | 0.0004 |
| 26:08:01 | 4 | 0.0006 | 51:07:01 | 31 | 0.0044 | 04:03:01 | 2 | 0.0003 |
| 31:08 | 3 | 0.0004 | 58:01:01 | 29 | 0.0041 | 04:04:01 | 2 | 0.0003 |
| 01:02:01 | 2 | 0.0003 | 50:01:01 | 26 | 0.0037 | 07:185 | 2 | 0.0003 |
| 02:30:01 | 2 | 0.0003 | 39:06:02 | 22 | 0.0031 | 12:98 | 2 | 0.0003 |
| 29:72 | 2 | 0.0003 | 35:08:01 | 19 | 0.0027 | 15:06:01 | 2 | 0.0003 |
| 69:01:01 | 2 | 0.0003 | 45:01:01 | 16 | 0.0023 | 03:54 | 1 | 0.0001 |
| 01:01:33 | 1 | 0.0001 | 47:01:01 | 13 | 0.0019 | 04:01:10 | 1 | 0.0001 |
| 02:02:01 | 1 | 0.0001 | 07:05:01 | 11 | 0.0016 | 05:16 | 1 | 0.0001 |
| 02:17:02 | 1 | 0.0001 | 18:03:01 | 10 | 0.0014 | 07:02:10 | 1 | 0.0001 |
| 02:38 | 1 | 0.0001 | 15:17:01 | 9 | 0.0013 | 07:75 | 1 | 0.0001 |
| 02:64:01 | 1 | 0.0001 | 15:24:01 | 9 | 0.0013 | 07:76:01 | 1 | 0.0001 |
| 03:01:14 | 1 | 0.0001 | 07:04:01 | 8 | 0.0011 | 08:03:01 | 1 | 0.0001 |
| 11:01:79 | 1 | 0.0001 | 57:03:01 | 7 | 0.0010 | 12:16:01 | 1 | 0.0001 |
| 24:03:01 | 1 | 0.0001 | 14:01:01 | 6 | 0.0009 | 15:09:01 | 1 | 0.0001 |
| 26:15 | 1 | 0.0001 | 44:27:01 | 5 | 0.0007 | 15:13:01 | 1 | 0.0001 |
| 33:05:01 | 1 | 0.0001 | 46:01:01 | 4 | 0.0006 | 16:15:01 | 1 | 0.0001 |
|  |  |  | 53:01:01 | 4 | 0.0006 | 16:201 | 1 | 0.0001 |
|  |  |  | 15:18:01 | 3 | 0.0004 |  |  |  |
|  |  |  | 15:39:01 | 3 | 0.0004 |  |  |  |
|  |  |  | 35:01:29 | 3 | 0.0004 |  |  |  |
|  |  |  | 39:06:01 | 3 | 0.0004 |  |  |  |
|  |  |  | 40:06:01 | 3 | 0.0004 |  |  |  |
|  |  |  | 48:01:01 | 3 | 0.0004 |  |  |  |
|  |  |  | 57:02:01 | 3 | 0.0004 |  |  |  |
|  |  |  | 07:07:01 | 2 | 0.0003 |  |  |  |
|  |  |  | 07:10:01 | 2 | 0.0003 |  |  |  |
|  |  |  | 13:01:01 | 2 | 0.0003 |  |  |  |
|  |  |  | 15:08:01 | 2 | 0.0003 |  |  |  |
|  |  |  | 15:16:01 | 2 | 0.0003 |  |  |  |
|  |  |  | 15:190:01 | 2 | 0.0003 |  |  |  |
|  |  |  | 39:24:01 | 2 | 0.0003 |  |  |  |
|  |  |  | 40:01:02 | 2 | 0.0003 |  |  |  |
|  |  |  | 40:12:01 | 2 | 0.0003 |  |  |  |
|  |  |  | 40:94 | 2 | 0.0003 |  |  |  |
|  |  |  | 44:03:02 | 2 | 0.0003 |  |  |  |
|  |  |  | 44:48:01 | 2 | 0.0003 |  |  |  |
|  |  |  | 51:08:01 | 2 | 0.0003 |  |  |  |
|  |  |  | 08:262 | 1 | 0.0001 |  |  |  |
|  |  |  | 15:01:10 | 1 | 0.0001 |  |  |  |
|  |  |  | 15:09:01 | 1 | 0.0001 |  |  |  |
|  |  |  | 15:34:01 | 1 | 0.0001 |  |  |  |
|  |  |  | 15:73:01 | 1 | 0.0001 |  |  |  |
|  |  |  | 27:03 | 1 | 0.0001 |  |  |  |
|  |  |  | 27:07:01 | 1 | 0.0001 |  |  |  |
|  |  |  | 27:12:01 | 1 | 0.0001 |  |  |  |
|  |  |  | 39:68 | 1 | 0.0001 |  |  |  |
|  |  |  | 40:01:20 | 1 | 0.0001 |  |  |  |
|  |  |  | 40:32 | 1 | 0.0001 |  |  |  |
|  |  |  | 42:01:01 | 1 | 0.0001 |  |  |  |
|  |  |  | 42:02:01 | 1 | 0.0001 |  |  |  |
|  |  |  | 44:04 | 1 | 0.0001 |  |  |  |
|  |  |  | 47:02 | 1 | 0.0001 |  |  |  |
|  |  |  | 51:01:19 | 1 | 0.0001 |  |  |  |
|  |  |  | 51:05:01 | 1 | 0.0001 |  |  |  |
|  |  |  | 52:01:02 | 1 | 0.0001 |  |  |  |

**Supplementary table 2.** HLA class I double homozygous haplotypes identified in this study (n = 153)

| haplotype | count |
| --- | --- |
| A*02:01:01 / 03:01:01 - B*07:02:01 / 07:02:01 - C*07:02:01 / 07:02:01 | 29 |
| A*03:01:01 / 11:01:01 - B*07:02:01 / 07:02:01 - C*07:02:01 / 07:02:01 | 10 |
| A*03:01:01 / 24:02:01 - B*07:02:01 / 07:02:01 - C*07:02:01 / 07:02:01 | 8 |
| A*02:01:01 / 02:01:01 - B*13:02:01 / 57:01:01 - C*06:02:01 / 06:02:01 | 6 |
| A*02:01:01 / 11:01:01 - B*07:02:01 / 07:02:01 - C*07:02:01 / 07:02:01 | 4 |
| A*03:01:01 / 11:01:01 - B*35:01:01 / 35:01:01 - C*04:01:01 / 04:01:01 | 3 |
| A*01:01:01 / 02:01:01 - B*13:02:01 / 13:02:01 - C*06:02:01 / 06:02:01 | 2 |
| A*02:01:01 / 02:01:01 - B*15:01:01 / 35:03:01 - C*04:01:01 / 04:01:01 | 2 |
| A*02:01:01 / 02:01:01 - B*15:01:01 / 40:01:01 - C*03:04:01 / 03:04:01 | 2 |
| A*02:01:01 / 03:01:01 - B*13:02:01 / 13:02:01 - C*06:02:01 / 06:02:01 | 2 |
| A*02:01:01 / 03:01:01 - B*40:01:01 / 40:01:01 - C*03:04:01 / 03:04:01 | 2 |
| A*02:01:01 / 11:01:01 - B*15:01:01 / 15:01:01 - C*04:01:01 / 04:01:01 | 2 |
| A*02:01:01 / 24:02:01 - B*13:02:01 / 13:02:01 - C*06:02:01 / 06:02:01 | 2 |
| A*02:01:01 / 25:01:01 - B*08:01:01 / 08:01:01 - C*07:01:01 / 07:01:01 | 2 |
| A*02:01:01 / 26:01:01 - B*07:02:01 / 07:02:01 - C*07:02:01 / 07:02:01 | 2 |
| A*02:01:01 / 30:01:01 - B*13:02:01 / 13:02:01 - C*06:02:01 / 06:02:01 | 2 |
| A*03:01:01 / 26:01:01 - B*35:01:01 / 35:01:01 - C*04:01:01 / 04:01:01 | 2 |
| A*03:01:01 / 31:01:02 - B*39:01:01 / 39:01:01 - C*12:03:01 / 12:03:01 | 2 |
| A*03:01:01 / 32:01:01 - B*07:02:01 / 07:02:01 - C*07:02:01 / 07:02:01 | 2 |
| A*11:01:01 / 26:01:01 - B*07:02:01 / 07:02:01 - C*07:02:01 / 07:02:01 | 2 |
| A*01:01:01 / 02:01:01 - B*08:01:01 / 08:01:01 - C*07:01:01 / 07:01:01 | 1 |
| A*01:01:01 / 02:01:01 - B*35:03:01 / 35:03:01 - C*04:01:01 / 04:01:01 | 1 |
| A*01:01:01 / 02:01:01 - B*40:01:01 / 40:01:01 - C*03:04:01 / 03:04:01 | 1 |
| A*01:01:01 / 02:01:01 - B*57:01:01 / 57:01:01 - C*06:02:01 / 06:02:01 | 1 |
| A*01:01:01 / 23:01:01 - B*44:03:01 / 44:03:01 - C*04:01:01 / 04:01:01 | 1 |
| A*01:01:01 / 24:02:01 - B*07:02:01 / 07:02:01 - C*07:02:01 / 07:02:01 | 1 |
| A*01:01:01 / 24:02:01 - B*13:02:01 / 13:02:01 - C*06:02:01 / 06:02:01 | 1 |
| A*02:01:01 / 02:01:01 - B*07:02:01 / 15:01:01 - C*03:04:01 / 03:04:01 | 1 |
| A*02:01:01 / 02:01:01 - B*07:02:01 / 39:01:01 - C*07:02:01 / 07:02:01 | 1 |
| A*02:01:01 / 02:01:01 - B*08:01:01 / 18:01:01 - C*07:01:01 / 07:01:01 | 1 |
| A*02:01:01 / 02:01:01 - B*15:01:01 / 15:01:01 - C*03:03:01 / 04:01:01 | 1 |
| A*02:01:01 / 02:01:01 - B*15:01:01 / 15:01:01 - C*03:04:01 / 04:01:01 | 1 |
| A*02:01:01 / 02:01:01 - B*27:05:02 / 27:05:02 - C*01:02:01 / 02:02:02 | 1 |
| A*02:01:01 / 02:01:01 - B*27:05:02 / 51:01:01 - C*01:02:01 / 01:02:01 | 1 |
| A*02:01:01 / 02:01:01 - B*27:05:02 / 56:01:01 - C*01:02:01 / 01:02:01 | 1 |
| A*02:01:01 / 02:01:01 - B*38:01:01 / 39:01:01 - C*12:03:01 / 12:03:01 | 1 |
| A*02:01:01 / 02:01:01 - B*44:02:01 / 44:02:01 - C*03:03:01 / 07:04:01 | 1 |
| A*02:01:01 / 02:01:01 - B*44:02:01 / 44:02:01 - C*05:01:01 / 07:04:01 | 1 |
| A*02:01:01 / 02:01:01 - B*44:05:01 / 57:01:01 - C*02:02:02 / 02:02:02 | 1 |
| A*02:01:01 / 02:01:01 - B*51:01:01 / 51:01:01 - C*02:02:02 / 15:02:01 | 1 |
| A*02:01:01 / 03:01:01 - B*15:01:01 / 15:01:01 - C*03:03:01 / 03:03:01 | 1 |
| A*02:01:01 / 03:01:01 - B*18:01:01 / 18:01:01 - C*12:03:01 / 12:03:01 | 1 |
| A*02:01:01 / 03:01:01 - B*44:02:01 / 44:02:01 - C*07:04:01 / 07:04:01 | 1 |
| A*02:01:01 / 03:01:01 - B*56:01:01 / 56:01:01 - C*01:02:01 / 01:02:01 | 1 |
| A*02:01:01 / 11:01:01 - B*40:02:01 / 40:02:01 - C*02:02:01 / 02:02:01 | 1 |
| A*02:01:01 / 24:02:01 - B*07:02:01 / 07:02:01 - C*07:02:01 / 07:02:01 | 1 |
| A*02:01:01 / 24:02:01 - B*15:01:01 / 15:01:01 - C*03:03:01 / 03:03:01 | 1 |
| A*02:01:01 / 26:01:01 - B*13:02:01 / 13:02:01 - C*06:02:01 / 06:02:01 | 1 |
| A*02:01:01 / 26:01:01 - B*15:01:01 / 15:01:01 - C*03:03:01 / 03:03:01 | 1 |
| A*02:01:01 / 26:01:01 - B*27:05:02 / 27:05:02 - C*01:02:01 / 01:02:01 | 1 |
| A*02:01:01 / 26:01:01 - B*38:01:01 / 38:01:01 - C*12:03:01 / 12:03:01 | 1 |
| A*02:01:01 / 26:01:01 - B*40:01:01 / 40:01:01 - C*03:04:01 / 03:04:01 | 1 |
| A*02:01:01 / 31:01:02 - B*13:02:01 / 13:02:01 - C*06:02:01 / 06:02:01 | 1 |
| A*02:01:01 / 31:01:02 - B*40:01:01 / 40:01:01 - C*03:04:01 / 03:04:01 | 1 |
| A*02:01:01 / 32:01:01 - B*40:02:01 / 40:02:01 - C*02:02:02 / 02:02:02 | 1 |
| A*02:01:01 / 68:01:01 - B*40:01:01 / 40:01:01 - C*03:04:01 / 03:04:01 | 1 |
| A*02:01:01 / 68:01:02 - B*15:01:01 / 15:01:01 - C*03:04:01 / 03:04:01 | 1 |
| A*02:01:01 / 68:01:02 - B*35:03:01 / 35:03:01 - C*04:01:01 / 04:01:01 | 1 |
| A*02:01:01 / 68:01:02 - B*40:01:01 / 40:01:01 - C*03:04:01 / 03:04:01 | 1 |
| A*03:01:01 / 03:01:01 - B*07:02:01 / 07:02:01 - C*07:02:01 / 07:185:01 | 1 |
| A*03:01:01 / 03:01:01 - B*07:02:01 / 07:04:01 - C*07:02:01 / 07:02:01 | 1 |
| A*03:01:01 / 03:01:01 - B*08:01:01 / 47:01:01 - C*07:01:01 / 07:01:01 | 1 |
| A*03:01:01 / 03:01:01 - B*18:01:01 / 35:03:01 - C*12:03:01 / 12:03:01 | 1 |
| A*03:01:01 / 11:01:01 - B*56:01:01 / 56:01:01 - C*01:02:01 / 01:02:01 | 1 |
| A*03:01:01 / 24:02:01 - B*35:01:01 / 35:01:01 - C*04:01:01 / 04:01:01 | 1 |
| A*03:01:01 / 24:02:01 - B*40:02:01 / 40:02:01 - C*02:02:02 / 02:02:02 | 1 |
| A*03:01:01 / 25:01:01 - B*35:01:01 / 35:01:01 - C*04:01:01 / 04:01:01 | 1 |
| A*03:01:01 / 26:01:01 - B*40:01:01 / 40:01:01 - C*03:04:01 / 03:04:01 | 1 |
| A*03:01:01 / 30:01:01 - B*07:02:01 / 07:02:01 - C*07:02:01 / 07:02:01 | 1 |
| A*03:01:01 / 68:01:02 - B*07:02:01 / 07:02:01 - C*07:02:01 / 07:02:01 | 1 |
| A*03:01:14 / 31:01:02 - B*07:02:01 / 07:02:01 - C*07:02:01 / 07:02:01 | 1 |
| A*11:01:01 / 11:01:01 - B*15:01:01 / 51:01:01 - C*04:01:01 / 04:01:01 | 1 |
| A*11:01:01 / 11:01:01 - B*35:01:01 / 44:03:01 - C*04:01:01 / 04:01:01 | 1 |
| A*11:01:01 / 24:02:01 - B*07:02:01 / 07:02:01 - C*07:02:01 / 07:02:01 | 1 |
| A*11:01:01 / 25:01:01 - B*56:01:01 / 56:01:01 - C*01:02:01 / 01:02:01 | 1 |
| A*11:01:01 / 26:01:01 - B*40:02:01 / 40:02:01 - C*02:02:02 / 02:02:02 | 1 |
| A*11:01:01 / 31:01:02 - B*51:01:01 / 51:01:01 - C*15:02:01 / 15:02:01 | 1 |
| A*11:01:01 / 68:01:02 - B*07:02:01 / 07:02:01 - C*07:02:01 / 07:02:01 | 1 |
| A*24:02:01 / 24:02:01 - B*07:02:01 / 39:06:02 - C*07:02:01 / 07:02:01 | 1 |
| A*24:02:01 / 24:02:01 - B*08:01:01 / 39:01:01 - C*07:02:01 / 07:02:01 | 1 |
| A*24:02:01 / 24:02:01 - B*13:02:01 / 37:01:01 - C*06:02:01 / 06:02:01 | 1 |
| A*24:02:01 / 25:01:01 - B*18:01:01 / 18:01:01 - C*12:03:01 / 12:03:01 | 1 |
| A*25:01:01 / 30:01:01 - B*13:02:01 / 13:02:01 - C*06:02:01 / 06:02:01 | 1 |
| A*25:01:01 / 31:01:02 - B*40:01:01 / 40:01:01 - C*03:04:01 / 03:04:01 | 1 |
| A*26:01:01 / 32:01:01 - B*38:01:01 / 38:01:01 - C*12:03:01 / 12:03:01 | 1 |

**Supplementary table 3**. *HLA-A*, *HLA-B* and *HLA-C* triple homozygous fibroblasts derived in this study.

| sample | HLA-A | HLA-B | HLA-C |
| --- | --- | --- | --- |
| SD2 | 25:01:01 | 18:01:01 | 12:03:01 |
| SD4 | 01:01:01 | 08:01:01 | 07:01:01 |
| SD5 | 03:01:01 | 56:01:01 | 01:02:01 |
| SD6 | 02:01:01 | 13:02:01 | 06:02:01 |
| SD7 | 03:01:01 | 35:03:01 | 04:01:01 |
| SD8 | 02:01:01 | 40:01:01 | 03:04:01 |
| SD9 | 02:01:01 | 13:02:01 | 06:02:01 |
| SD10 | 02:01:01 | 40:01:01 | 03:04:01 |
| SD11 | 03:01:01 | 35:01:01 | 04:01:01 |
| SD14 | 25:01:01 | 18:01:01 | 12:03:01 |
| SD15 | 03:01:01 | 07:02:01 | 07:02:01 |
| SD16 | 02:01:01 | 40:01:01 | 03:04:01 |
| SD17 | 03:01:01 | 07:02:01 | 07:02:01 |
| SD19 | 02:01:01 | 44:02:01 | 05:01:01 |
| SD20 | 02:01:01 | 27:05:02 | 02:02:02 |
